# Conservative aortic valve surgery and supra-coronary tube: a case report

**DOI:** 10.1093/jscr/rjad504

**Published:** 2023-10-14

**Authors:** Abdel Malick Idrissa, Hicham Wazaren, Hanae Bouhdadi, Briki Jihad, Saadouni Youssef, Chakib Benlafqih, Jaafar Rhissassi, Rochde Sayah, Mohammed Laaroussi

**Affiliations:** Department of Cardiovascular Surgery A of Ibn Sina University Hospital Center, Mohammed V University of Rabat, Rabat 10170, Morocco; Department of Cardiovascular Surgery A of Ibn Sina University Hospital Center, Mohammed V University of Rabat, Rabat 10170, Morocco; Department of Cardiovascular Surgery A of Ibn Sina University Hospital Center, Mohammed V University of Rabat, Rabat 10170, Morocco; Department of Cardiovascular Surgery A of Ibn Sina University Hospital Center, Mohammed V University of Rabat, Rabat 10170, Morocco; Department of Cardiovascular Surgery A of Ibn Sina University Hospital Center, Mohammed V University of Rabat, Rabat 10170, Morocco; Department of Cardiovascular Surgery A of Ibn Sina University Hospital Center, Mohammed V University of Rabat, Rabat 10170, Morocco; Department of Cardiovascular Surgery A of Ibn Sina University Hospital Center, Mohammed V University of Rabat, Rabat 10170, Morocco; Department of Cardiovascular Surgery A of Ibn Sina University Hospital Center, Mohammed V University of Rabat, Rabat 10170, Morocco; Department of Cardiovascular Surgery A of Ibn Sina University Hospital Center, Mohammed V University of Rabat, Rabat 10170, Morocco

**Keywords:** conservative surgery, aortic valve, supracoronary tube, commissary points

## Abstract

Conservative aortic valve surgery is becoming an effective alternative to aortic valve replacement for patients with aortic insufficiency (AI) and ascending aortic aneurysms. It has become a crucial component of the therapeutic arsenal recommended by the learned societies. This paper reports the case of a patient with severe AI following dilatation of the supra-coronary aorta that underwent aortic plasty associated with a supra-coronary tube in the Cardiovascular Surgery Department ‘A’ of the Ibn Sina Hospital, Rabat, Morocco.

## Introduction

Conservative aortic valve surgery was developed to preserve the aortic valve in patients with severe aortic insufficiency (AI) on a healthy valve, whether associated or not with an aneurysm of the ascending aorta. M. Yacoub described conservative aortic valve surgery first, followed by T. David [1]. Since then, this conservative aortic valve surgery has been improved with new procedures and anatomical areas. In expert hands, the surgical outcomes are outstanding.

Thus, this article reports the case of a young patient who underwent conservative aortic surgery for severe AI on a healthy aortic valve, following a dilatation of the supra-coronary aorta.

## Clinical presentation

Mr. N. H. is 42 years old, hypertensive and being treated for hypertensive nephropathy complicated by chronic end-stage renal disease.

He was diagnosed with dyspnea stages II and III of the NYHA with worsening of his lower limb edemas.

The clinical examination was done on a conscious patient, well oriented in time and space, with a general condition corresponding to stage 2 of the WHO Performance Status.

On auscultation, the cardiovascular examination revealed regular heart sounds as well as a diastolic murmur in the aortic and pulmonary foci. Peripheral pulses, which are ample and bouncy, are perceived symmetrically. It is vital to indicate the presence of the following right signs: edemas of the lower limbs, hepatomegaly and jugular veins turgidity.

The vesicular murmur was detected on pleuropulmonary examination in both pulmonary fields with basithoracic crackling rales.

**Figure 1:**
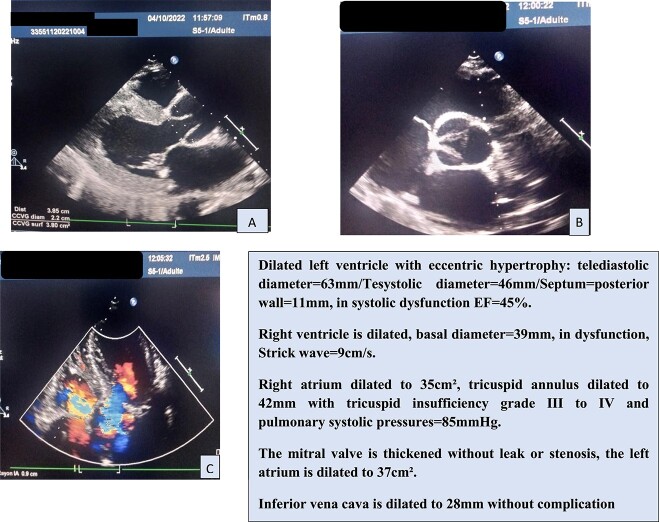
(A–C) Transthoracic echocardiogrphy sections.

In view of this clinical picture, transthoracic echocardiography (TTE) was performed, which confirmed the significant AI and ascending aorta dilatation (Fig. 1).

**Figure 2:**
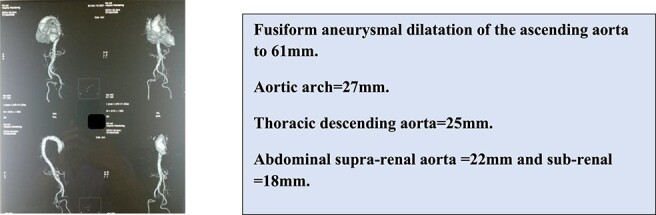
Thoracic angioscanner showing.

A thoracic angioscanner was used to complete the assessment in front of the dilatation of the ascending aorta (Fig. 2).

The aneurysmal dilatation is only on the supratubal aorta, preserving the aortic annulus and the sinus of valsalva. This is a type 1 IA that causes traction on the cusps at the level of the STJ, resulting in central diastasis and aortic leakage.

**Figure 3:**
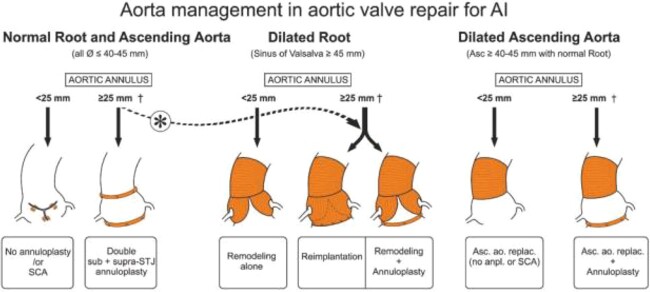
Picture from literature of diagram of aorta management.

The indication was granted to execute a double aortic and tricuspid plasty as well as a supracoronary tube (Fig. 3).

**Figure 4:**
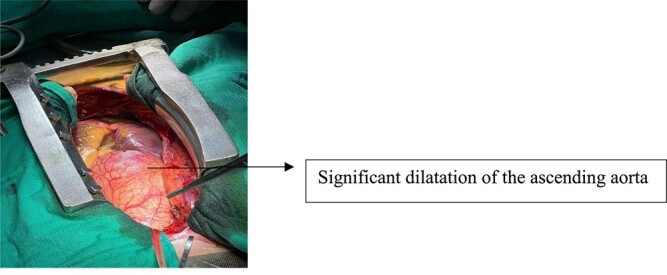
Cardiac structures after sternotomy.

The patient was taken to the surgical table. A vertical median sternotomy was performed under general anesthesia and in dorsal decubitus. Dilated heart chambers and a large ascending aorta were observed after pericardiotomy (Fig. 4).

An extracorporeal circulation cannula is installed between a femoral arterial cannula and two laced cava cannulas.

Cardiac arrest is produced through blood cardioplegia combined with local cooling with crushed ice.

**Figure 5:**
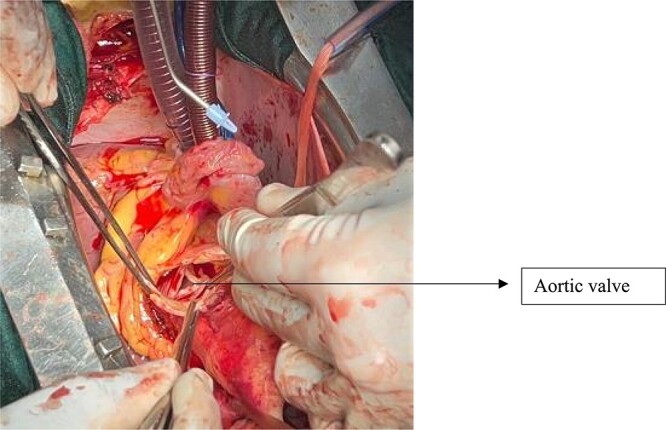
Aortic valve after resection of the supra-coronary aorta.

A low horizontal and subsequently a high vertical aortotomy are used to expose the aortic valve while simultaneously removing the aneurysmal part of the supracoronary aorta. The aortic valve has thin cusps of normal height with a central diastasis and no calcification on the body of the cusps or at the commissures (Fig. 5).

**Figure 6:**
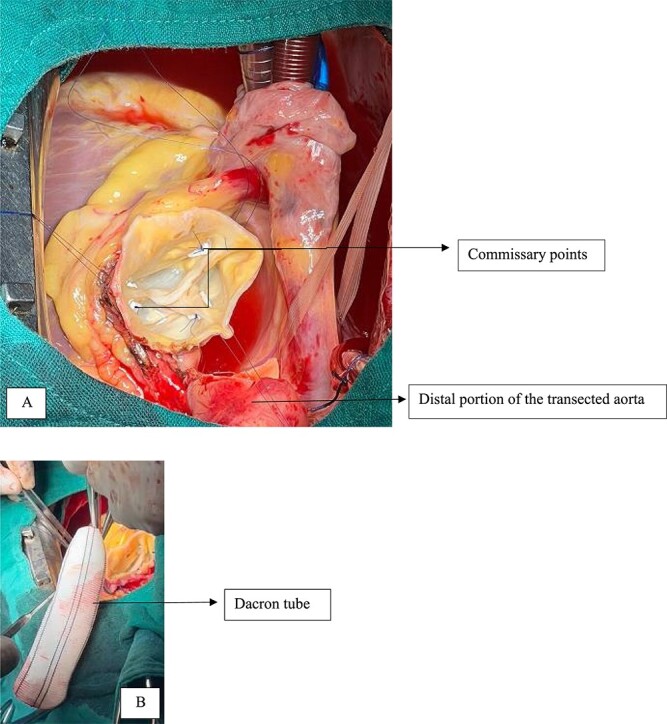
Commissural points at the three commissures, disappearance of the central diastasis.

After the valve is examined, we commence the aortic plasty by creating commissural sutures with 4/0 pledged prolene at the level of each commissure to achieve good cusp coaptation (Fig. 6a). A bulb test is performed to ensure that the aortic valve is properly sealed. The proximal anastomosis of the aorta is then accomplished using a dacron tube and 4/0 prolene. An autologous pericardium patch is used to strengthen this anastomosis. The distal anastomosis with 4/0 prolene completes the aortic plasty (Fig. 6b).

In addition, the patient underwent tricuspid valve annuloplasty using the Devega procedure.

**Figure 7:**
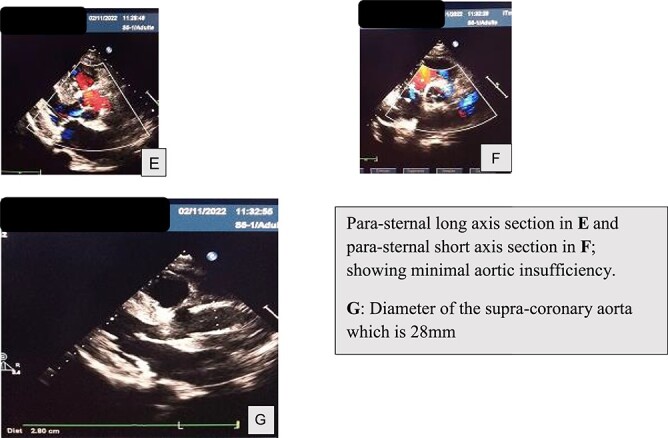
Postoperative TTE findings.

An echocardiographic check-up was conducted on day 1 and day 10 postoperatively, which confirmed the tightness of the aortic valve and the success of the aortic plasty (Fig. 7).

## Discussion

Aortic plastic surgery is a challenging procedure for surgeons since it seeks to correct aortic root dystrophy while taking into account the complexities of its hemodynamics and physiology. Yacoub was the first to pioneer conservative aortic valve surgery with his Remodeling approach [1], and David was the second with his Reimplantation technique [2]. Since then, numerous strategies have emerged that aim to standardize management by presenting a technique based on the phenotypic of aortic root dystrophy.

When available, intra-operative transesophageal echocardiography is used to guide valve restorations at our institution. As a result, the competence of a valve is also evaluated indirectly.

The decision to repair (rather than replace) when the outcome will not be seen by echocardiography until post-operative day 1 merits more debate in terms of patient selection and intra-operative assessment of valve competency.

Currently, aortic regurgitation of all etiologies can theoretically be repaired with acceptable results. However, freedom from reoperation for type III lesions seems to be inferior to other types. Furthermore, use of a pericardial patch was identified as a risk factor for recurrence. In terms of patient selection and intra-operative assessment of valve competency, the decision to proceed with repair (vs replacement) warrants more consideration.

Patients who are candidates for conservative aortic valve surgery at our institution are chosen based on the following preoperative criteria: LV end-diastolic diameter of 70 mm prior to deterioration of its function, tricuspid aortic valve geometric height (GH) of 18 mm, nonrheumatic etiology as cutoff values and items to perform aortic valve plasty. Concerning the intra-operative evaluation Type Ia AR is typically identified by examining the middle straight jet due to poor cusp coaptation caused by tethering despite adequate GH and eH. Trans-esophageal echocardiography is still the best way to assess the efficacy of an aortic valvuloplasty during surgery.

In most publications, the long-term outcome of aortic plasty is outstanding. Lansac E *et al*. [1] report that in centers experienced in employing the David procedure, the rate of patients free of reoperation can reach 96% after a 20-year follow-up. Aortic plasty performed on central AI (type 1) or prolapse (type 2) lasts longer than AI performed on cusp retraction (type 3). The plasty’s durability is the same whether the valve is tricuspid or bicuspid.

Piccinini *et al*. report a 91% survival rate without re-intervention after 10 years [3].

Furthermore, we find that patients who have had an aortic plasty have a higher quality of life than those who have had a Bentall [4].

Despite encouraging results, aortic plastic surgery is still limited to a few hospitals with a high volume of valve surgery. This demonstrates how difficult it is to perform aortic plastic surgery.

## Conclusion

Aortic plasty provides a stable long-term result with less morbidity at the cost of a careful patient selection and an adaptation of the surgical approach to the phenotype of aortic root dystrophy.

## Data Availability

Data sharing is not applicable to this article as no datasets were generated or analyzed during the current study.
